# Dim light at night induces cardiac injury in zebrafish embryos via disrupted chloride homeostasis

**DOI:** 10.1016/j.isci.2026.115796

**Published:** 2026-06-03

**Authors:** Risi Chen, Ying Zeng, Meixin Min, Ke Xu, Jia Wang, Tingting Liu, Yan Zhao, Weixin Zhang, Shuting Cheng, Yiyue Zhang, Chunru Wang, Dan Deng, Xiaoping Xiao

**Affiliations:** 1 Ganzhou Key Laboratory for Drug Screening and Discovery, School of Geography and Environmental Engineering, Gannan Normal University, Ganzhou 341000, China; 2 Jiangxi Provincial Key Laboratory of Synthetic Pharmaceutical Chemistry, Gannan Normal University, Ganzhou 341000, China; 3 Shenzhen Center for Disease Control and Prevention, Shenzhen 518055, China; 4 Health Ministry Key Laboratory of Chronobiology, West China School of Basic Medical Sciences & Forensic Medicine, Sichuan University, Chengdu 610041, China; 5 Innovation Centre of Ministry of Education for Development and Diseases, School of Medicine, South China University of Technology, Guangzhou 510006, China; 6 Beijing National Research Center for Molecular Sciences, Key Laboratory of Molecular Nanostructure and Nanotechnology, Institute of Chemistry, Chinese Academy of Science, Beijing 100190, China; 7 Gannan Health Vocational College, Ganzhou 341000, China

**Keywords:** environmental health, molecular biology

## Abstract

Artificial light at night (ALAN) is an escalating environmental stressor linked to cardiovascular disease, yet its underlying mechanisms remain poorly understood. Here, we investigate the molecular basis of this pathology using zebrafish embryos for mechanistic discovery and murine models for translational validation under a chronic dim- light- at -night (dLAN) paradigm. dLAN exposure induced cardiac injury characterized by pericardial edema and hemodynamic impairment. Transcriptomic profiling revealed dysregulation of inflammatory and oxidative stress pathways, accompanied by marked suppression of the calcium-activated chloride channel accessory protein *zclca1*. Genetic knockdown of *zclca1* recapitulated core dLAN pathologies, while pharmacological intervention with the chloride-modulating diuretic bumetanide reversed cardiac dysfunction in both species. These findings identify a conserved “dLAN-CLCA-chloride-cardiac injury” axis, wherein light pollution disrupts chloride homeostasis to drive cardiac pathology, suggesting that chloride modulation may represent a potential therapeutic strategy for mitigating light pollution-induced cardiovascular injury.

## Highlights

• DLAN induces cardiac injury in zebrafish embryos and mice
• dLAN suppresses *clca1*, causing hyperchloremia and chloride dysregulation
• *zclca1* knockdown recapitulates dLAN-induced cardiac pathology
• Bumetanide rescues cardiac dysfunction across species via chloride modulation

## Graphical abstract

**Figure:**
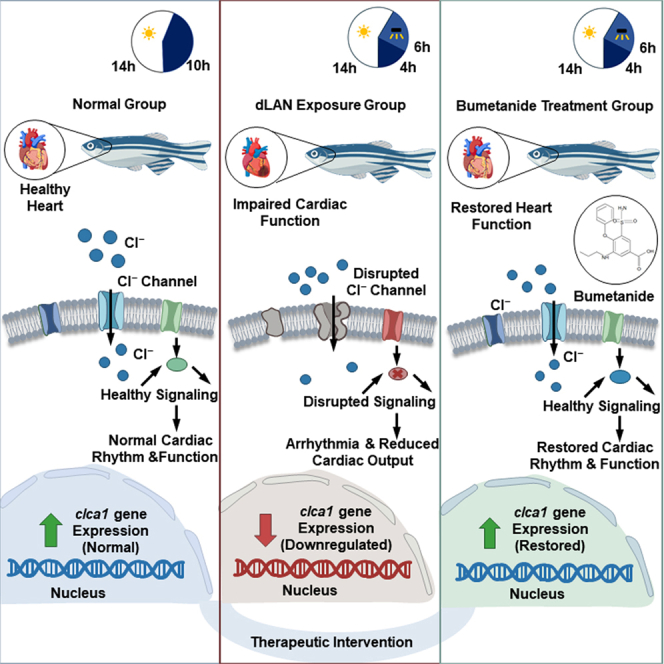

## Introduction

Artificial light at night (ALAN) is an escalating byproduct of urbanization that impinges on over 83% of the global population and poses an emerging cardiovascular risk.^1^^,^^2^ Longitudinal epidemiological studies have consistently linked chronic exposure to dim light at night with increased incidence of hypertension and adverse cardiac outcomes in human populations.^3^^,^^4^ Furthermore, in mammals, dLAN disrupts circadian rhythms primarily by suppressing melatonin secretion, leading to autonomic nervous system imbalance, metabolic dysregulation, and cardiovascular dysfunction, thereby elevating the risk of cardiac diseases.^5^^,^^6^^,^^7^ Large-scale population studies have shown that higher nighttime light exposure is independently associated with increased incidence of coronary heart disease, myocardial infarction, heart failure, atrial fibrillation, and stroke.^8^ Animal experiments further confirm that dLAN dampens the circadian amplitude of heart rate and blood pressure, exhibiting sex-specific patterns in autonomic regulation. Intervention studies suggest that restricting feeding windows can partially restore cardiovascular rhythms, indicating that behavioral adjustments may mitigate the adverse effects of nocturnal light exposure.^9^

Critically, the experimental paradigm employed in this study was specifically designed to model the intrusion of light upon the core biological night—a key feature of real-world ALAN exposure-rather than simply extending the photoperiod. This distinction is chronobiologically significant, as dLAN and photoperiod extension can elicit distinct physiological consequences.

In developing zebrafish embryos, which serve as our primary model for discovery, light information is transduced not only visually but also through non-visual opsins (e.g., Opn4a). These opsins can influence local circadian clocks and downstream processes, including cardiogenesis, though the precise pathways relaying photic information to the embryonic heart remain an active area of investigation.^10^^,^^11^ Given these fundamental differences in photoperiodic information transmission across evolutionary lineages (e.g., fish, birds, and mammals),^12^^,^^13^ we employed a cross-species validation strategy-using zebrafish for mechanistic discovery and mice for translational confirmation-to ensure the identified pathway’s broader relevance.

Despite the established clinical correlation between dysregulated chloride homeostasis—both hypochloremia and hyperchloremia—and worsened cardiac remodeling and contractile dysfunction in heart failure patients,^14^^,^^15^ a direct molecular link between ALAN and chloride imbalance remains elusive. The calcium-activated chloride channel accessory (CLCA) protein family is a pivotal regulator of chloride transport,^16^ yet it is unknown whether environmental disruptors like dLAN impair CLCA function to causally drive cardiac injury.

To bridge this knowledge gap, we employed a tiered, cross-species strategy. We leveraged the optical transparency and genetic tractability of transgenic zebrafish embryos for real-time cardiac phenotyping and initial mechanistic discovery. Subsequently, key findings and therapeutic interventions were rigorously validated in a murine model to assess translational relevance to mammals, thereby mitigating the inherent limitations of direct extrapolation from fish to humans. We hypothesized that dLAN downregulates CLCA proteins, leading to hyperchloremia, pericardial edema, and cardiac dysfunction—a pathway amenable to targeted pharmacotherapy. Therefore, this study is centered on mechanistic discovery-identifying how dLAN disrupts chloride homeostasis to induce cardiac injury-rather than modeling a specific adult disease. This approach allows us to isolate a conserved pathogenic pathway across species.

## Results

### dLAN induces structural and functional cardiac injury in zebrafish embryos

**Figure 1:**
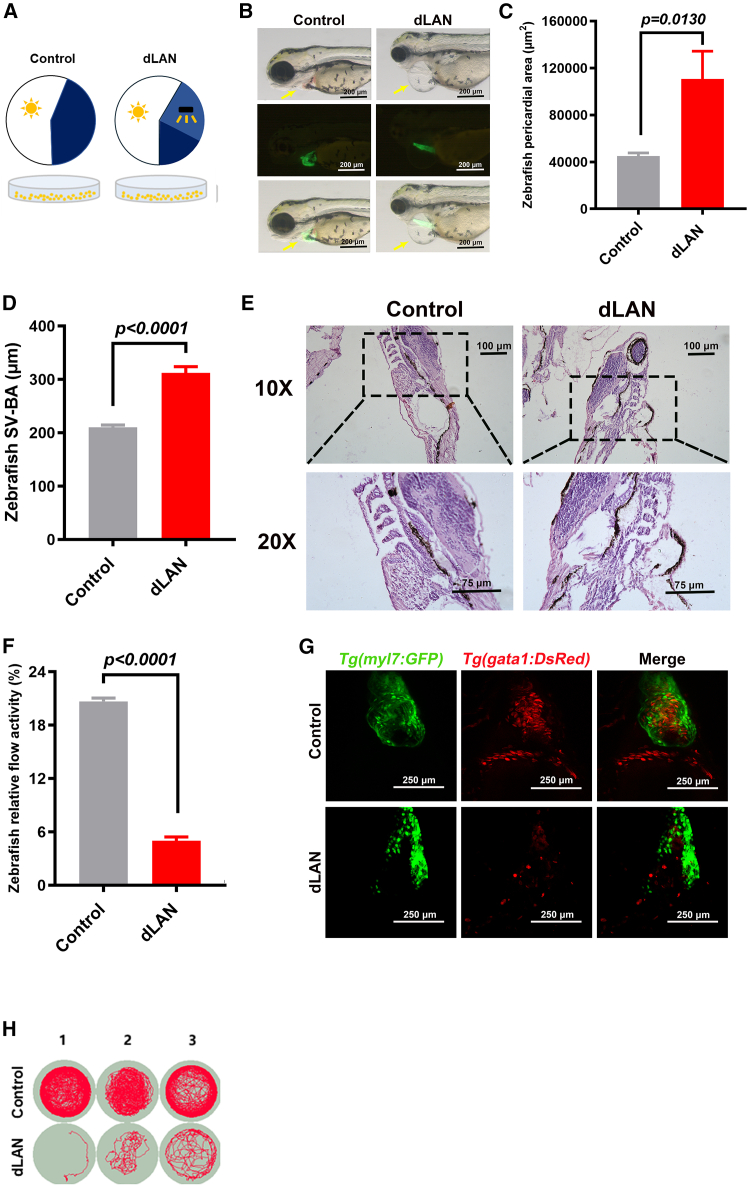
dLAN induces structural and functional cardiac injury in zebrafish embryos (A) Schematic of the experimental dLAN paradigm. Control embryos were maintained under a standard 14 h light/10 h dark cycle (lights on: 8:00 a.m. to 10:00 p.m.). The dLAN group received 14 h of standard light, followed by 6 h of dim light (2.25 lux; 10:00 p.m. to 4:00 a.m.) and 4 h of complete darkness (4:00 a.m. to 8:00 a.m.), compressing the dark phase. Exposure began at 6 hpf and lasted for 72 h. (B) Representative fluorescence images of Tg*(myl7:EGFP)* zebrafish embryos hearts (green: myocardium). Yellow arrows indicate pericardial edema (fluid accumulation) and ventricular malformation. Scale bars, 200 μm. (C) Quantification of pericardial area (μm^2^) showing significant expansion in dLAN-exposed embryos (*n* = 10 zebrafish embryos/group). (D) Increased sinus venosus-bulbus arteriosus (SV-BA) distance (μm), indicating impaired cardiac linearization and chamber alignment (*n* = 10 zebrafish embryos/group). (E) Hematoxylin and eosin (H&E)-stained heart sections revealing disrupted cardiomyocyte parallel alignment in dLAN-exposed embryos. Scale bars, 75 μm (bottom) and 100 μm (top). (F) Reduced hemodynamic activity in Tg*(gata1:DsRed)* embryos (*n* = 12 zebrafish embryos/group). (G) Confocal images of Tg*(myl7:EGFP;gata1:DsRed)* hearts showing diminished intracardiac erythrocyte retention (red) within the myocardium (green). Scale bars, 250 μm. (H) Locomotor activity heatmaps over 10 min for three representative individual zebrafish embryos per condition (Control [LD] and dLAN). The heatmaps depict repeated measurements of mobility for each individual, demonstrating a consistent suppression of locomotor activity in dLAN-exposed embryos compared to LD controls. (C, D, and F) Data are presented as mean ± SEM. Statistical significance was determined by unpaired two-tailed Student’s *t* test.

To determine whether dLAN exposure induces cardiac damage in zebrafish, similar to its effects in mammals,^17^^,^^18^ we specifically examined low-intensity dLAN (<5 lux) at 2.25 lux (Figure 1A), which simulates typical nightlight conditions. Beyond triggering pericardial edema, dLAN caused systemic developmental abnormalities, including swim bladder agenesis and body axis curvature (Figures S1A and S1B). For cardiac-specific assessment, we utilized Tg*(myl7:EGFP)* zebrafish with GFP-labeled myocardium. dLAN-exposed fish exhibited pronounced pericardial edema (Figure 1B), enlarged pericardial area (Figure 1C), and structural defects, notably elongated sinus venosus-bulbus arteriosus (SV-BA) distance (Figure 1D). Histopathological analysis via hematoxylin and eosin (H&E) staining further confirmed aberrant cardiomyocyte alignment (Figure 1E).

In Tg*(gata1:DsRed)* zebrafish embryos with erythrocyte-specific fluorescence, dLAN significantly reduced hemodynamic activity (Figure 1F) and disrupted blood flow patterns (Figure S2 and Videos S1A and S1B). To directly evaluate cardiac pumping efficiency, double-transgenic Tg*(myl7*:*EGFP*;*gata1*:*DsRed)* zebrafish embryos enabled real-time visualization of intracardiac erythrocyte distribution, revealing markedly diminished erythrocyte retention (Figure 1G). Consistent with known correlations between cardiac dysfunction and locomotor impairment,^19^^,^^20^ dLAN-exposed zebrafish embryos showed significantly reduced locomotor capacity (Figure 1H), independent of acetylcholinesterase (AChE) activity (Figure S3). Collectively, these results establish that dLAN exposure induces both structural and functional cardiac impairments in zebrafish embryos.

Video S1A. dLAN impairs cardiac hemodynamics in Tg(gata1:dsRed) zebrafish, related to Figure 1 Physiological erythrocyte circulation in control zebrafish under standard light-dark cycle.

Video S1B. dLAN impairs cardiac hemodynamics in Tg(gata1:dsRed) zebrafish, related to Figure 1 Compromised ventricular ejection and reduced intracardiac erythrocyte retention in dLAN-exposed larvae.

### Multi-pathway mechanisms drive cardiac dysfunction under dLAN

**Figure 2:**
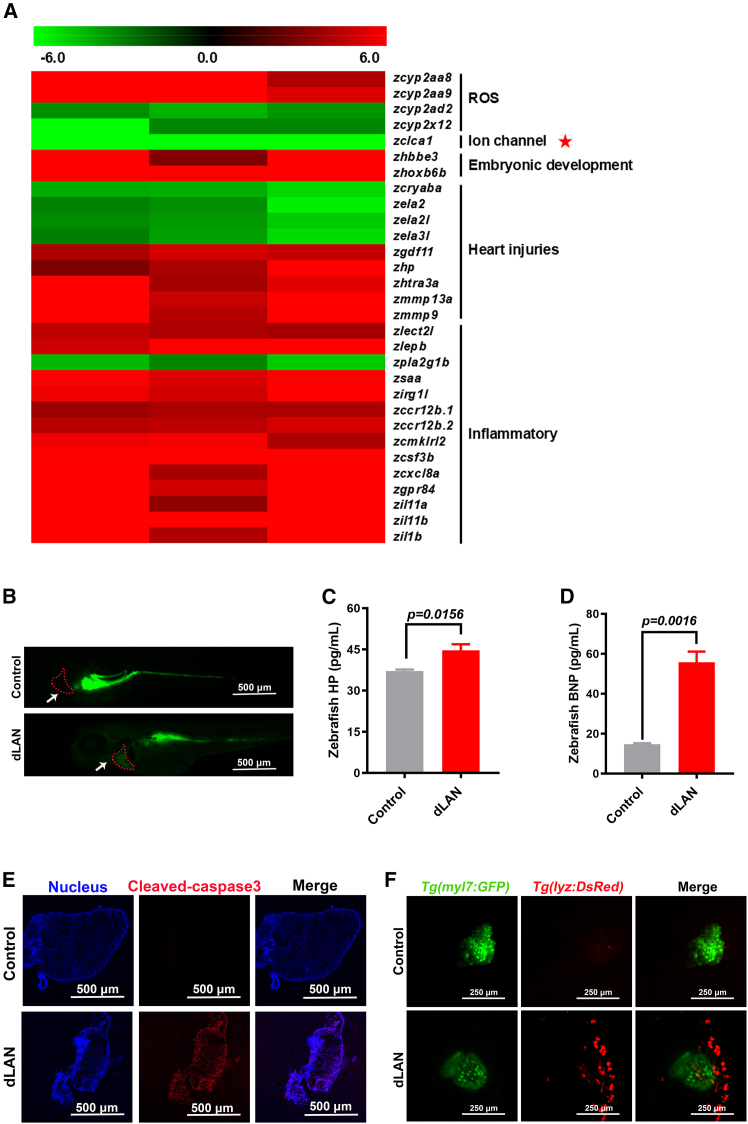
Multi-pathway dysregulation drives dLAN-induced cardiac injury (A) Heatmap of RNA-seq analysis comparing dLAN-exposed and control zebrafish embryos (*n* = 5 zebrafish embryos/group). (B) DCFH-DA staining showing elevated reactive oxygen species (ROS) in dLAN-exposed hearts (dashed outline: cardiac region). Scale bars, 500 μm. (C and D) ELISA quantification of serum heart failure biomarkers, including haptoglobin (HP) (C) and B-type natriuretic peptide (BNP)(D), both significantly increased in dLAN-exposed embryos (*n* = 3–4 pools of 50 zebrafish embryos/group). (E) Immunofluorescence staining for activated caspase-3 (red) and DAPI (blue) confirming enhanced apoptosis in dLAN-exposed hearts. Scale bars, 500 μm. (F) Confocal images of Tg*(myl7:EGFP;lyz:DsRed)* hearts showing neutrophil infiltration (red) into the pericardial space (green myocardium) in dLAN-exposed embryos. Scale bars, 250 μm. (C and D) Data are presented as mean ± SEM. Statistical significance was determined by unpaired two-tailed Student’s *t* test.

To elucidate the impact of dLAN on cardiac function, we performed transcriptomic sequencing (RNA seqencing, RNA-seq). Kyoto encyclopedia of genes and genomes (KEGG) pathway analysis of the same dataset revealed widespread dysregulation of multiple pathological pathways, including significant enrichment of reactive oxygen species (ROS)-generating genes, cardiac disease markers (including *zsaa* and *zhp*, orthologs of human heart failure biomarkers), and inflammation-related genes (Figure 2A). Consistent with transcriptomic findings, functional validation confirmed elevated cardiac ROS levels through 2',7'-Dichlorodihydrofluorescein diacetate (DCFH-DA) staining (Figure 2B). Serum analysis further demonstrated increased heart failure biomarkers-haptoglobin (HP) and B-type natriuretic peptide (BNP)-via enzyme-linked immunosorbent assay (ELISA) (Figures 2C and 2D). Concurrently, upregulated cardiac fibrosis-related genes (*zMMP9*, *zMMP13a*) and downregulation of cardioprotective *zebrafish cryaba* (*zcryaba*) suggested enhanced cardiomyocyte apoptotic susceptibility, which was corroborated by immunofluorescence staining showing increased apoptosis (Figure 2E). Neutrophil-specific imaging in Tg*(lyz:DsRed)* zebrafish embryos revealed pronounced inflammatory infiltration, aligning with transcriptomic signatures (Figure 2F). However, cardiac developmental regulators (*znkx2.5*, *zhand2*, *zgata4*, *zbmp4*, *zmyh6*) remained unaltered (Table S1), indicating that dLAN-induced pathology operates through multifactorial stress mechanisms independent of developmental interference. Crucially, analysis of core circadian clock genes revealed no significant transcriptional alterations at this embryonic developmental stage (Table S1), suggesting distinct initial mechanisms from those in mature animals.

### CLCA1-mediated chloride dysregulation is the core pathogenic driver

**Figure 3:**
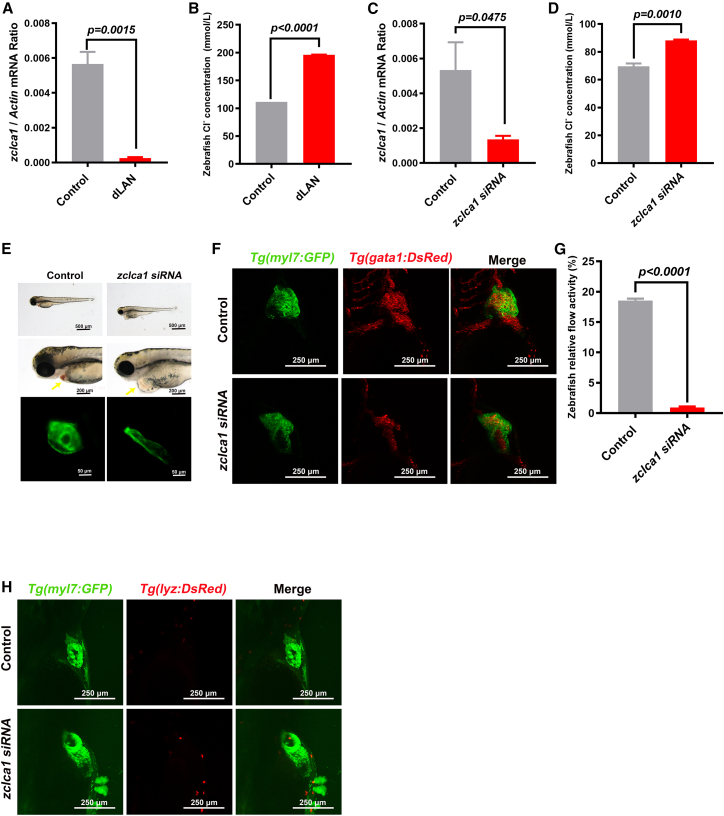
CLCA1 suppression mediates chloride dysregulation and cardiac injury (A) qPCR validation of *zclca1* mRNA downregulation in dLAN-exposed zebrafish embryos (*n* = 3 pools of 5 zebrafish embryos/group). (B) Serum chloride ion concentration showing hyperchloremia in dLAN-exposed embryos (*n* = 3 pools of 20 zebrafish embryos/group). (C) qPCR confirming efficient *zclca1* knockdown (KD) via siRNA microinjection (*n* = 4 pools of 5 zebrafish embryos/group). (D) *zclca1* KD-induced hyperchloremia (*n* = 3 pools of 50 zebrafish embryos/group). (E) Tg*(myl7:EGFP)* images showing pericardial edema (yellow arrows) in *zclca1* KD embryos, phenocopying dLAN injury. Scale bars, 50 μm (bottom), 200 μm (middle), and 500 μm (top). (F) Reduced intracardiac erythrocyte retention in Tg*(myl7:EGFP;gata1:DsRed)* hearts after *zclca1* KD. Scale bars, 250 μm. (G) Impaired hemodynamic activity in *zclca1* KD embryos (*n* = 10 zebrafish embryos/group). (H) Enhanced neutrophil recruitment (red) in Tg*(myl7:EGFP;lyz:DsRed)* hearts after *zclca1* KD. Scale bars, 250 μm. (A–D and G) Data are presented as mean ± SEM. Statistical significance was determined by unpaired two-tailed Student’s *t* test.

Given the established link between chloride imbalance and pericardial edema, and the central role of chloride channels in homeostasis, we investigated the potential involvement of the calcium-activated chloride channel accessory (CLCA) family. Our transcriptomic profiling revealed a pronounced downregulation of *zclca1* in dLAN-exposed embryos (Figure 2A), which was validated by quantitative real-time PCR (qPCR) to be nearly complete (Figure 3A). This transcriptional suppression was associated with systemic hyperchloremia, indicated by a marked elevation in serum chloride levels (Figure 3B). To assess the functional role of *zclca1* beyond transcriptional correlation, we performed genetic knockdown using specific small interfering RNA (siRNA). This loss-of-function approach achieved efficient mRNA silencing (Figure 3C) and successfully recapitulated the hyperchloremia observed under dLAN (Figure 3D). Notably, *zclca1* knockdown reproduced the comprehensive spectrum of dLAN-induced cardiac injuries, including pericardial edema, structural abnormalities (Figure 3E), reduced intracardiac erythrocyte retention (Figure 3F), hemodynamic compromise (Figures 3G and S4, and Videos S2A and S2B), and enhanced neutrophil infiltration (Figure 3H). The specificity of this phenotype was corroborated by the unaltered expression of key cardiac developmental regulators (Figure S5), indicating that the effects were not attributable to nonspecific developmental disruption. Taken together, these findings demonstrate that CLCA1 is essential for chloride homeostasis and that its suppression is sufficient to drive the cardiac injury phenotype observed in dLAN-exposed zebrafish embryos.

Video S2A. zclca1 knockdown phenocopies dLAN-induced contractile dysfunction, related to Figure 3 Normal systolic-diastolic coordination in scramble siRNA-injected controls.

Video S2B. zclca1 knockdown phenocopies dLAN-induced contractile dysfunction, related to Figure 3 Impaired ventricular emptying efficiency and hemodynamic disturbance following *zclca1* silencing.

### Bumetanide rescues injury via chloride homeostasis restoration

**Figure 4:**
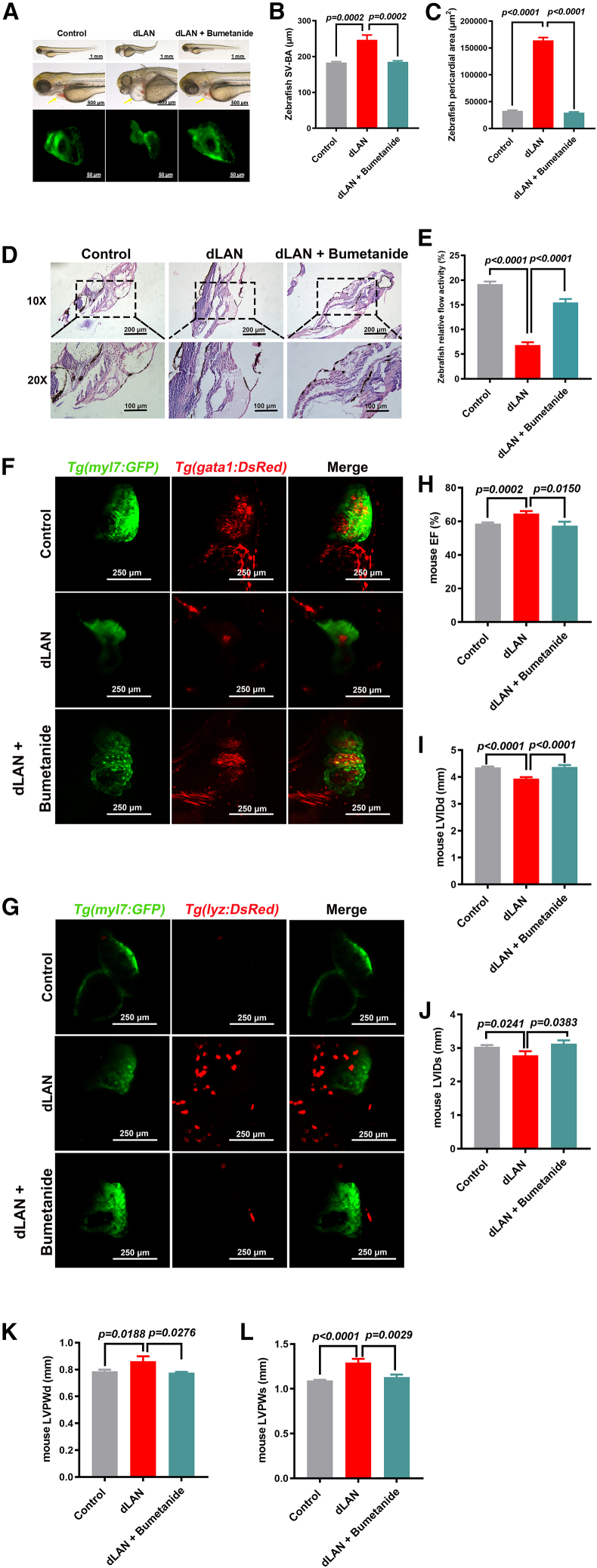
Bumetanide rescues cardiac injury by restoring chloride homeostasis (A) Tg*(myl7:EGFP)* images showing amelioration of pericardial edema (yellow arrows) after 24 h bumetanide treatment (5 nM) in dLAN-exposed embryos. Scale bars, 50 μm (bottom), 500 μm (middle), and 1,000 μm (top). (B and C) Therapeutic efficacy as evidenced by reduced SV-BA distance (μm) (B) and pericardial area (μm^2^) (C) post-bumetanide (*n* = 10 zebrafish embryos/group). (D) H&E-stained sections demonstrating restored cardiomyocyte alignment after bumetanide. Scale bars, 100 μm (bottom), and 200 μm (top). (E) Improved hemodynamic activity in bumetanide-treated Tg*(gata1:DsRed)* embryos (*n* = 20 zebrafish embryos/group). (F) Restored intracardiac erythrocyte retention in Tg*(myl7:EGFP;gata1:DsRed)* hearts post-bumetanide. Scale bars, 250 μm. (G) Attenuated neutrophil infiltration in bumetanide-treated Tg*(myl7:EGFP;lyz:DsRed)* hearts. Scale bars, 250 μm. (H–L) Echocardiographic parameters in dLAN-exposed mice showing that bumetanide (10 mg/kg/day, oral) improved ejection fraction (EF; %) (H), left ventricular internal diameter (LVIDd/s; mm) (I and J), and posterior wall thickness (LVPWd/s; mm) (K–L) (*n* = 4–8 mice/group). Statistical significance was determined by unpaired *t* test with Bonferroni correction for multiple comparisons across five parameters and two contrasts (dLAN vs. control; dLAN+bumetanide vs. dLAN), yielding an adjusted significance threshold of *p < 0.005*. After correction, dLAN vs. control remained significant for EF (*p = 0.0002*), LVIDd (*p < 0.0001*), and LVPWs (*p < 0.0001*); bumetanide treatment significantly improved LVIDd (*p < 0.0001*) and LVPWs (*p = 0.0029*), while EF showed a strong trend toward improvement (*p = 0.015*). LVIDs and LVPWd exhibited consistent directional trends but did not reach statistical significance after correction. All data are presented as mean ± SEM. (B, C, and E) Data are presented as mean ± SEM. Statistical significance was determined by one-way ANOVA followed by Tukey’s post hoc test.

Given that pericardial edema mechanically compromises cardiac function by elevating intrapericardial pressure and restricting ventricular filling, we sought therapeutic strategies to alleviate dLAN-induced cardiac pathology. Based on our previous findings implicating oxidative stress, inflammation, and chloride dysregulation, we selected three pharmacological agents with distinct mechanisms, including the antioxidant N-acetyl-L-cysteine (NAC), the nuclear factor κB (NF-κB) and calcium signaling inhibitor 4‑N‑[2‑(4‑phenoxyphenyl) ethyl]quinazoline‑4,6‑diamine (QNZ, EVP4593), and the loop diuretic bumetanide, which directly targets chloride transport via the Na^+^-K^+^-2Cl^−^ cotransporter (NKCC). This selection allowed us to test whether specifically correcting chloride imbalance would be uniquely therapeutic. Administration of these compounds to dLAN-exposed embryos with established edema revealed that only bumetanide produced a significant rescue, effectively alleviating pericardial edema (Figures 4A and S6). Treatment with bumetanide consistently improved multiple hallmarks of injury, as evidenced by a reduced SV-BA distance (Figure 4B), a diminished pericardial area (Figure 4C), restored cardiomyocyte alignment (Figure 4D), enhanced hemodynamic activity (Figures 4E and S7, and Videos S3A, S3B, and S3C), increased intracardiac erythrocyte retention (Figure 4F), and attenuated cardiac inflammation (Figure 4G). To translationally validate the chloride homeostasis mechanism identified in zebrafish, we extended our investigation to a murine model, adopting a hypothesis-driven approach focused on key functional readouts-echocardiographic parameters (ejection fraction [EF], left ventricular dimension [LVID]) and serum chloride levels-to directly assess cardiac performance and ion imbalance. Consistent with our zebrafish findings, bumetanide administration ameliorated cardiac dysfunction in dLAN-exposed mice, significantly improving EF, LVIDd/s, and posterior wall thickness (LVPWd/s) (Figures 4H–4L). These cross-species results not only confirm the conserved role of chloride dysregulation in dLAN-induced injury but also provide preliminary evidence that bumetanide may have potential in mitigating light pollution-induced cardiac damage through restoration of chloride homeostasis.

Video S3A. Bumetanide rescues dLAN-induced hemodynamic abnormalities, related to Figure 4 Baseline hemodynamic profile in untreated control zebrafish.

Video S3B. Bumetanide rescues dLAN-induced hemodynamic abnormalities, related to Figure 4 Pathological blood flow stasis and reduced ejection fraction in dLAN-exposed larvae.

Video S3C. Bumetanide rescues dLAN-induced hemodynamic abnormalities, related to Figure 4 Restored ventricular ejection kinetics and erythrocyte circulation after bumetanide treatment.

### Evolutionary conservation of CLCA-dependent cardiac injury

**Figure 5:**
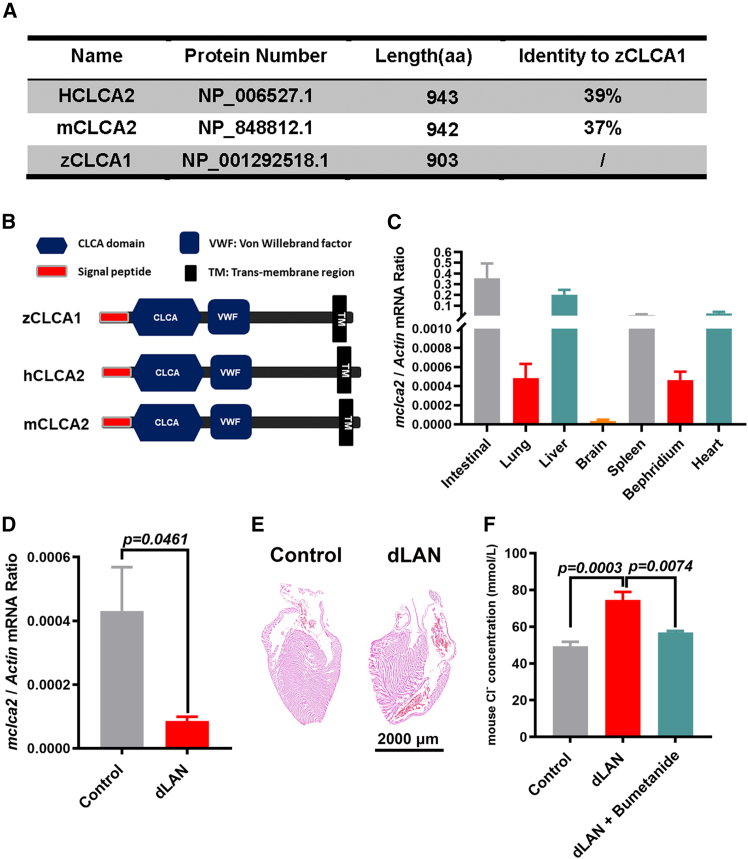
Evolutionary conservation of CLCA-dependent cardiac injury (A and B) Bioinformatics comparison and functional module analysis of CLCA1 in humans, mice, and zebrafish. (A) Percentage amino acid identity among human (hCLCA2), mouse (mCLCA2), and zebrafish (zCLCA1). (B) Schematic diagrams of hCLCA2, mCLCA2, and zCLCA1 proteins. Functional modules were predicted using the SMART (http://smart.embl-heidelberg.de/) and Pfam (http://pfam.xfam.org/) websites. (C) qPCR analysis of *mclca2* tissue distribution in mice. (D) dLAN-induced downregulation of cardiac *mclca2* mRNA in mice (*n* = 4 mice/group). (E) H&E-stained murine heart sections showing myocardial lesions after dLAN exposure. Scale bars 2,000 μm. (F) Serum chloride levels showed that bumetanide reversed dLAN-induced hyperchloremia in mice (*n* = 4–8 mice/group). (C, D, and F) Data are presented as mean ± SEM. Statistical significance was determined by unpaired two-tailed Student’s *t* test.

To assess the translational relevance of our findings, we investigated the conservation of the CLCA-dependent injury axis. Bioinformatics analysis revealed high evolutionary conservation of critical functional domains among zebrafish zCLCA1, murine mCLCA2, and human hCLCA2 (Figures 5A and 5B), suggesting conserved roles in chloride homeostasis regulation. In mice, quantitative PCR confirmed substantial *mclca2* expression in cardiac tissue, in addition to other organs (Figure 5C). Crucially, chronic dLAN exposure in mice recapitulated the core pathology observed in zebrafish, inducing significant downregulation of cardiac *mclca2* expression (Figure 5D) and provoking visible myocardial lesions (Figure 5E). This conserved CLCA suppression was functionally linked to a shared physiological outcome, as evidenced by the significant hyperchloremia in dLAN-exposed mice that was effectively normalized by bumetanide treatment (Figure 5F). These results demonstrate that the fundamental mechanism of dLAN-induced injury-centered on CLCA suppression, chloride dysregulation, and resultant cardiac dysfunction-is evolutionarily conserved from zebrafish to mammals. This functional homology not only underscores the broad relevance of light pollution as a cardiovascular risk factor but also validates the utility of the zebrafish embryo as a discovery model for identifying conserved pathogenic pathways and evaluating potential therapeutics.

## Discussion

Our findings reveal a conserved molecular mechanism through which dLAN disrupts cardiac homeostasis, independent of developmental or adult-specific pathologies. Collectively, our data support a pathogenic axis, wherein dLAN-induced suppression of CLCA expression impairs chloride conductance, leading to extracellular chloride accumulation, increased osmotic load, and ultimately culminating in pericardial edema and cardiac dysfunction. This mechanistic focus clarifies the direct causal link between environmental light exposure and cardiac dysfunction. Our study establishes that chronic exposure to a light-at-night paradigm-designed to simulate the intrusion of artificial light into the core biological night-induces cardiac injury through a conserved “CLCA-chloride-pericardial edema” axis. We demonstrate that this exposure downregulates CLCA proteins in both zebrafish embryos and mammals, triggering hyperchloremia, pericardial edema, and myocardial dysfunction-a pathology that is reversible by the loop diuretic bumetanide.

A key advance of this work is the identification of CLCA suppression as a critical molecular event linking dLAN to chloride imbalance and cardiac injury. The sufficiency of CLCA suppression to drive pathology was demonstrated by genetic knockdown of *zclca1* in zebrafish, which recapitulated the hyperchloremia, edema, and functional deficits observed under dLAN. These results provide causal evidence beyond correlation and help explain clinical observations linking hyperchloremia to adverse cardiac outcomes.^15^ Although chloride homeostasis *in vivo* is subject to complex systemic regulation-including renal and neuroendocrine processes -not fully addressed here;- the efficacy of chloride-targeting therapy with bumetanide across species underscores the pathophysiological relevance of this ion imbalance.

Notably, the “CLCA-chloride-cardiac injury” axis appears to operate independently of the core circadian transcriptional machinery in embryonic zebrafish, unlike in more mature animals, where dLAN robustly disrupts clock gene expression.^21^ This developmental distinction highlights that light pollution can harm the developing heart through mechanisms that precede or bypass canonical circadian disruption, emphasizing the particular vulnerability of early cardiovascular systems to environmental insults.

Evolutionary conservation further strengthens the translational relevance of our findings. High structural homology among CLCA orthologs across species, along with bumetanide’s efficacy in both zebrafish and mice, supports the use of zebrafish embryos as a powerful discovery model for environmental cardiotoxicity. Moreover, the conservation of this injury pathway underscores light pollution as a universal health threat, with urgent public health implications given that over 83% of the global population is exposed to ALAN^1^—a risk factor correlated with cardiovascular disease in human studies.^4^

Our experimental design intentionally distinguished between the intrusion of light into the biological night and a simple extension of photoperiod- a chronobiologically meaningful difference. The cardiac pathology induced under our dLAN paradigm, which preserved low light intensity but compressed the dark phase, confirms that the timing of light exposure is a critical determinant of its pathological impact, independent of total day length.

Mechanistically, the failure of antioxidants to rescue cardiac edema—despite confirmed ROS elevation^22^^,^^23^—contrasts with the efficacy of bumetanide, suggesting that chloride dysregulation plays a primary role in this pathology. However, the benefits of bumetanide likely derive from a combination of reduced cardiac preload (via diuresis) and modulation of chloride homeostasis, rather than chloride correction alone. The observed changes in CLCA expression may also reflect adaptive responses to systemic hemodynamic alterations, a complexity that merits further investigation.

Translationally, bumetanide’s dual actions-alleviating fluid overload and attenuating inflammation-highlight its potential relevance for further translational investigation. However, its repurposing for light pollution-induced cardiac effects requires careful consideration. Future studies should define optimal dosing, monitor side effects such as electrolyte imbalances, and identify vulnerable populations-such as shift workers, who experience both high ALAN exposure and elevated cardiovascular risk.^4^ Although hyperchloremia is common in acute heart failure,^15^ the chronic use of bumetanide for environmental prophylaxis remains to be established.

### Limitations of the study

This study has several limitations. First, we used broad-spectrum white light, and thus the potential spectral-specific effects of different light wavelengths on CLCA suppression and cardiac injury remain undefined. Second, while we establish a central role for CLCA-mediated chloride dysregulation, chloride homeostasis *in vivo* is systemically regulated by renal and neuroendocrine processes not fully captured in our embryonic model. Third, the sample size in the murine bumetanide-treatment subgroup was relatively small (*n* = 4), which, despite revealing a significant therapeutic trend, necessitates future validation with larger cohorts. Finally, the pleiotropic effects of bumetanide, including its potent diuretic and potential anti-inflammatory actions, mean that its benefits may not be solely attributable to chloride correction. These limitations highlight important avenues for future research but do not detract from the core finding of a conserved CLCA-chloride pathway in dLAN-induced cardiac injury. Additionally, the use of the same light intensity across species does not account for differences in photoreception and photoperiodic responses between zebrafish and mice, which may modulate the observed effects.

## Resource availability

### Lead contact

Further information and requests for resources and reagents should be directed to and will be fulfilled by the lead contact, Xiaoping Xiao (xiaoxiaoping100@163.com).

### Materials availability

This study did not generate new unique reagents.

### Data and code availability

• This paper does not report original code.
• RNA-seq data have been deposited in the NCBI short read archive (SRA) and are publicly available as of the date of publication. Accession number is listed in the key resources table.
• Any additional information required to reanalyze the data reported in this paper is available from the lead contact upon request.

## Acknowledgments

This work was supported by grants from the 10.13039/501100001809National Natural Science Foundation of China (32260172), the NHC Key Laboratory of Chronobiology at 10.13039/501100004912Sichuan University (NHCC-2024-05), and the Jiangxi Provincial Double Thousand Talents Program (jxsq2023102009, jxsq2023102010).

## Author contributions

X.X. and R.C. designed the experiments; X.X. wrote the manuscript; R.C. performed majority of the experiments and analyzed data; Y.Z. helped with the animal experiment; J.W. and D.D. assisted in completing the microinjection of *zclca1 siRNA*; M.M., K.X., and T.L. helped with the RNA isolation and qPCR detection; Y.Z., W.Z., S.C., Y.Z., and C.W. contributed experimental suggestions and strengthened the writing of manuscript. All authors reviewed, critiqued and provided comments to the text.

## Declaration of interests

The authors declare no competing interests.

## Declaration of generative AI and AI-assisted technologies in the writing process

During the preparation of this work, the authors used DeepSeek in order to assist with language polishing, text formatting according to journal guidelines, and generating suggestions for key elements such as the title, abstract, and keywords. After using this tool/service, the authors reviewed and edited the content as needed and takes full responsibility for the content of the publication.

## STAR★Methods

### Key resources table

**Table:** 

REAGENT or RESOURCE	SOURCE	IDENTIFIER
**Antibodies**		

Cleaved-Caspase3 Rabbit Monoclonal Antibody	Beyotime Biotech Inc	Cat# AF1150
YF® 647 Goat Anti-Rabbit IgG (H&L)	Uelandy Bioscience	Cat# Y6109L
Chemicals		
DAPI	Uelandy Bioscience	Cat# D4080
Bumetanide	MedChemExpress	Cat# HY-17468
N-acetylcysteine	MedChemExpress	Cat# HY-110256
QNZ	MedChemExpress	Cat# HY-13812
Agarose, Low melting	Sangon Biotech	Cat#A600015

**Critical commercial assays**		

Ultrapure RNA Kit	Jiangsu CoWin Biotech Co., Ltd	Cat# CW0581M
HiFiScript gDNA Removal RT MasterMix	Jiangsu CoWin Biotech Co., Ltd	Cat# CW2020M
2× SuperFast Universal SYBR Master Mix	Jiangsu CoWin Biotech Co., Ltd	Cat# CW3888H
BNP ELISA Kits	Coibo Biotechnology	Cat# CB11080-Hu
HP ELISA Kits	Coibo Biotechnology	Cat# CB10809-Hu
Reactive oxygen species Assay Kit	Nanjing Jiancheng Bioengineering Institute	Cat# E004-1-1
Acetylcholinesterase (ACHE)	Nanjing Jiancheng Bioengineering Institute	Cat# H529-1-1
Chlorine Assay Kit	Nanjing Jiancheng Bioengineering Institute	Cat# C003-2-1

**Experimental models: Organisms/strains**		

C57BL/6 mice	Shenzhen center for disease control and prevention	N/A
Tg*(myl7:EGFP*) / Zebrafish	Gannan Normal University	N/A
Tg(*myl7:EGFP;gata1:DsRed*) / Zebrafish	Gannan Normal University	N/A
Tg(*gata1:DsRed*) / Zebrafish	Gannan Normal University	N/A
Tg(*myl7:EGFP;lyz:DsRed*) / Zebrafish	Gannan Normal University	N/A
*AB* / Zebrafish	Gannan Normal University	N/A

**Oligonucleotides**		

*zβ-actin*	FP: CCTTCCAGCAGATGTGGATTAG	N/A
*zβ-actin*	RP: TGAAGTGGTAACAGTCCGTTTAG	N/A
*zbmp4*	FP: CCCAGATCAAACAGGGGACC	N/A
*zbmp4*	RP: AGGTGTTGTGCCTCACCAAA	N/A
*zgata4*	FP: GCCGGGATACCGCAACTTAT	N/A
*zgata4*	RP: AGTACGGAGCTGTCGAAGTG	N/A
*zmyh6*	FP: GCTCCTTCCTCGGTGTGAA	N/A
*zmyh6*	RP: TTTTCAGACTCGGCGCTTT	N/A
*zhand2*	FP: GACGCCAAAGAAGAAAGGCG	N/A
*zhand2*	RP: TCAGCTCCAATGCCCAAACA	N/A
*znkx2.5*	FP: CAGGAGGACAAAGGCAAC	N/A
*znkx2.5*	RP: CTCTTCCTCTGCTTGGGC	N/A
*zclca1*	FP: CAAGTCTGGAAGCCGTGACC	N/A
*zclca1*	RP: AGGATGAGACAAACAGCCCG	N/A
*mβ-actin*	FP: CTGAGAGGGAAATCGTGCGT	N/A
*mβ-actin*	RP: CCACAGGATTCCATACCCAAGA	N/A
*mclca2*	FP: GGGCACTGGACTTACACGCTG	N/A
*mclca2*	RP: GCACCTGCTCCGCCATCCA	N/A
*Control siRNA*	Upper primer: UUCUCCGAACGUGUCACGUTT	N/A
*Control siRNA*	Lower primer: ACGUGACACGUUCGGAGAATT	N/A
*zclca1 siRNA*	Upper primer: CUCCAGACUUUCUCUUAAATT	N/A
*zclca1 siRNA*	Lower primer: UUUAAGAGAAAGUCUGGAGTT	N/A

**Software and algorithms**		

GraphPad Prism 8	Graphpad	https://www.graphpad.com/
Danio Vision	Noldus	https://noldus.com/
qRCR soft 4.0	Analytik jena	http://www.analytik-jena.com.cn/
DanioScope	Noldus	https://noldus.com/

**Deposited data**		

RNA-seq data	NCBI SRA	PRJNA1249967

### Experimental model and study participant details

#### Zebrafish (*Danio rerio*)

##### Species/strain

Zebrafish of the following lines were used: AB wild-type, Tg*(myl7:EGFP)* (myocardium-specific GFP expression), Tg*(gata1:DsRed)* (erythrocyte-specific DsRed expression), Tg*(myl7:EGFP;gata1:DsRed)* (double transgenic), and Tg*(myl7:EGFP;lyz:DsRed)* (double transgenic for myocardium and neutrophil visualization).

##### Age/developmental stage

Embryos were collected from natural spawning and experiments began at 6 hours post-fertilization (hpf).

##### Sex

Zebrafish embryos at 78 hpf do not exhibit differentiated gonads or secondary sexual characteristics; therefore, sex could not be determined at this developmental stage. Consequently, the influence of sex on the results could not be assessed. This represents a limitation of the study, as potential sex-specific responses to dLAN exposure during later developmental stages remain unknown.

##### Maintenance and care

Adult zebrafish were maintained in a recirculating aquaculture system at 28 ± 1°C under a 14-hour light/10-hour dark photoperiod and fed twice daily with brine shrimp. Embryos were obtained through natural spawning and cultivated in petri dishes under the same temperature conditions.

##### Experimental groups

Embryos were randomly assigned to control (standard 14L:10D cycle) or dim-light-at-night (dLAN) exposure groups. For pharmacological interventions, dLAN-exposed embryos with established pericardial edema were treated with bumetanide (5 nM), N-acetylcysteine (NAC), QNZ (EVP4593), or vehicle control (DMSO < 0.1%).

#### Mice (Mus musculus)

##### Species/strain

C57BL/6 mice.

##### Age/developmental stage

Eight-week-old mice at the start of the experiment; age at endpoint was approximately 12 weeks (30 days of exposure).

##### Sex

Both male and female mice were used, with equal sex distribution within each experimental group (n = 8 per group, 4 males and 4 females). This balanced design allows for assessment of sex-specific effects. However, the primary analyses in this study were not stratified by sex due to sample size limitations within subgroups (particularly after randomization for bumetanide treatment). While no overt sex-dependent differences were observed in the main phenotypes, the possibility of sex-specific influences cannot be excluded and warrants further investigation in larger cohorts.

##### Maintenance and care

Mice were housed in a specific pathogen-free (SPF) animal facility under standard individually ventilated caging (IVC) systems with sterile bedding, nesting materials, and enrichment items. Environmental conditions were strictly controlled: temperature 22 ± 1°C, relative humidity 50% ± 10%, under a 12-hour light/12-hour dark cycle (light phase: 7:00 AM to 7:00 PM). Mice had *ad libitum* access to irradiated standard laboratory rodent diet and autoclaved purified water. Bedding, feed, and water were regularly replenished, and cages were changed at least twice weekly.

##### Experimental groups

Mice were randomly divided into control (standard 12L:12D cycle) and dLAN-exposed groups (n = 8 per group, equal sex distribution). On day 23 of dLAN exposure, the dLAN group was further randomized into two subgroups: dLAN + vehicle (saline, oral gavage, n = 4) and dLAN + bumetanide (10 mg/kg/day, oral gavage, n = 4). Treatment continued for 7 days alongside continued dLAN exposure.

#### Ethics statement

All experimental procedures were performed in accordance with the Guidelines for Laboratory Animal Use established by the Experimental Animal Management Committee and approved by the Institutional Animal Care and Use Committee (gnnu2022-0628), Gannan Normal University, Jiangxi, China.

### Method details

#### Zebrafish husbandry and embryonic exposure

The transgenic zebrafish lines used in this study are all well-established strains maintained in our laboratory. Zebrafish were housed in a recirculating aquaculture system at 28 ± 1°C under a 14-hour light/10-hour dark photoperiod and fed twice daily with brine shrimp. Embryos were obtained through natural spawning of adult fish and cultivated in petri dishes.

To model the circadian-disruptive effects of environmentally relevant light pollution, we established a chronic dim-light-at-night (dLAN) exposure paradigm starting at 6 hours post-fertilization (hpf). This model was specifically designed to intrude upon the core biological night, rather than merely extend the photoperiod, thereby mimicking the circadian misalignment caused by artificial light at night (ALAN) in urban environments. The selected light intensity (2.25 lux of white light) represents common levels of residential ambient light pollution (e.g., from streetlights or electronic devices), which typically range from 1-10 lux indoors.^1^

At 6 hpf, zebrafish embryos were randomly assigned to either a control group or a dLAN exposure group. All embryos were maintained at 28.5 ± 1°C under a base photoperiod with lights on at 8:00 AM and off at 10:00 PM. The control group was kept under a standard 14-hour light/10-hour dark (14L:10D) cycle. In contrast, the experimental group was exposed to 14 hours of standard light followed by 6 hours of 2.25 lux light exposure (from 10:00 PM to 4:00 AM). Each night concluded with 4 hours of complete darkness (from 4:00 AM to 8:00 AM) before the cycle restarted. Both groups were maintained under these conditions for 72 hours at 28 ± 1°C.

#### Murine dLAN exposure

C57BL/6 mice were provided by the Shenzhen Center for Disease Control and Prevention. The mice were housed in a specific pathogen-free (SPF) animal facility. All animals were group-housed in standard individually ventilated caging (IVC) systems equipped with sterile bedding, nesting materials, and enrichment items for gnawing. Environmental conditions in the animal room were strictly controlled: temperature was maintained at 22 ± 1°C, relative humidity at 50% ± 10%, under a 12-hour light/12-hour dark cycle (light phase: 7:00 AM to 7:00 PM). The mice had *ad libitum* access to irradiated standard laboratory rodent diet and autoclaved purified water. Bedding, feed, and water were regularly replenished, and cages were changed at least twice weekly to maintain hygienic living conditions. All animal experimental procedures were conducted in accordance with approved guidelines from the Institutional Animal Care and Use Committee.

In mouse experiments, we implemented a corresponding dim light at night (dLAN) exposure paradigm. Eight-week-old C57BL/6 mice were randomly divided into control and dLAN-exposed groups (n = 8 per group, with equal sex distribution) and maintained under SPF conditions at 22 ± 1°C. The control group was maintained on a standard 12-hour light/12-hour dark cycle (light phase: 7:00 AM to 7:00 PM). The dLAN group, following the same 12-hour standard light phase, received 2.25 lux white light exposure during hours 2-8 of the dark phase (7:00 PM to 7:00 AM), specifically from 9:00 PM to 3:00 AM, to simulate environmental light pollution at night. Complete darkness was subsequently restored until the commencement of the next light phase. All lighting was provided by full-spectrum LED sources. The dLAN group underwent this exposure regimen continuously for 30 days, while the control group maintained the standard 12-hour light/12-hour dark circadian rhythm.

#### Pharmacological intervention

##### For zebrafish

Zebrafish embryos which exhibited significant pericardial edema following dLAN exposure were treated with a final concentration of 5 nM bumetanide. The control group received an equivalent volume of DMSO (final concentration below 1/1000). Cardiac morphology was dynamically monitored 24 hours post-treatment using a Zeiss microscope, with both still images and videos captured. Data analysis was performed using the DanioScope software.

##### For mice

On day 23 of dLAN exposure, the dLAN group was further randomized into two subgroups: one subgroup received bumetanide (10 mg/kg/day) via oral gavage at 9:00 AM daily, while the control subgroup received an equivalent volume of saline vehicle via the same route and schedule. Both subgroups continued dLAN exposure for an additional 7 days. After the intervention period, blood was collected via the tail vein to measure serum chloride ion levels, and cardiac function was assessed using echocardiography.

#### Measurement of zebrafish blood flow

In zebrafish blood flow measurement, the Tg(*gata1:DsRed*) transgenic line was used, which expresses red fluorescent protein in erythrocytes, and assessment was performed through a combination of *in vivo* fluorescence imaging and an automated analysis system. The specific procedure was as follows: transgenic embryos at 6 hpf were cultured under specific lighting conditions until the target developmental stage, and unanesthetized live zebrafish were immobilized in 1% low-melting-point agarose in glass-bottom confocal dishes. Fine needles were used to adjust the sample position so that the eyes overlapped and the trunk and tail fin were aligned in the same horizontal plane. Subsequently, using a fluorescence microscope with excitation/emission filter settings suitable for DsRed fluorescent protein, continuous videos of at least 5 seconds were recorded for both the cardiac region and the caudal vein blood flow. The acquired videos were imported into the DanioScope analysis system, and a standardized region of interest (ROI) was defined at a fixed segment of the caudal vein. The software automatically calculated the blood flow activity percentage by analyzing the dynamic changes in pixel intensity caused by erythrocyte passage through the vessel on a frame-by-frame basis. The calculation process first involved extracting the average pixel intensity of each frame within the ROI to form a signal sequence S(t), then computing the absolute difference in signal between adjacent frames ΔS(t), and further deriving the raw activity index based on the root mean square of all ΔS(t) values. Finally, through normalization with the maximum possible signal change value ΔS_max under the given conditions, the activity percentage reflecting blood flow intensity was generated.

#### RT-qPCR analysis

Total RNA was extracted using the RNA Kit (CW0581M). RNA purity was assessed by measuring the A260/A280 ratio (1.8-2.0) with a NanoDrop spectrophotometer, and integrity was verified by 1% agarose gel electrophoresis. Reverse transcription was performed using the HiFiScript gDNA Removal RT MasterMix (CW2020M) in a two-step reaction: genomic DNA was first removed by incubation at 42°C for 5 minutes, followed by cDNA synthesis using a mixture of Oligo(dT) and random hexamer primers.

Quantitative real-time PCR was carried out using 2× SuperFast Universal SYBR Master Mix on a QuantStudio 5 Real-Time PCR System. The 20 μL reaction mixture contained 10 μL SYBR Master Mix, 0.4 μL each of forward and reverse primers (final concentration 0.2 μM), 2 μL cDNA template, and 7.2 μL nuclease-free water. The amplification protocol consisted of an initial denaturation at 95°C for 30 seconds, followed by 40 cycles of denaturation at 95°C for 5 seconds and annealing/extension at 60°C for 30 seconds. Specificity of the amplification products was confirmed by melt curve analysis. The relative expression levels of target genes were calculated using the 2^(-ΔΔCt)^ method (qRCR soft 4.0), with *β-actin* (zebrafish) or *β-actin* (mice) serving as reference genes. The specific primer sequences used in this study are detailed in the STAR Methods section.

#### Zebrafish embryos whole-transcriptome sequencing

Total RNA was extracted from whole-body samples of zebrafish embryos exposed to ALAN or control conditions (5 per group) using TRIzol Reagent from Invitrogen . After DNase I treatment to remove genomic DNA contamination , RNA integrity was assessed using NanoDrop and Agilent 2100 Bioanalyzer . Strand-specific paired-end sequencing was performed by Novogene Corporation, with raw sequencing data subjected to FastQC quality control and adapter removal. The cleaned reads were aligned to the zebrafish reference genome (GRCz11) using SOAPaligner/SOAP2 (allowing ≤2 mismatches), and gene expression was quantified with featureCounts. Differential expression analysis was conducted using edgeR (v3.42.4) , with screening criteria set at |log2|≥1. All raw data have been deposited in the NCBI SRA database under accession number PRJNA1249967.

#### Immunofluorescence staining

Tissue samples were promptly dissected and placed in pre-cooled aluminum foil containers, embedded in optimal cutting temperature (OCT) compound, and rapidly frozen in isopentane pre-chilled with liquid nitrogen. The embedded tissue blocks were equilibrated in a -20°C cryostat and sectioned at a thickness of 5 μm. The intact sections were transferred onto glass slides and stored at -20°C for subsequent use.

The detailed immunofluorescence staining procedure was as follows: After equilibrating the slides to room temperature, OCT compound was washed off with PBS. The samples were then permeabilized with 0.1% Triton X-100 for 1 hour at room temperature to enhance cell membrane permeability. Following permeabilization, the solution was decanted and replaced directly with a blocking solution containing 5% BSA, incubated for 3–4 hours at room temperature. After blocking, the solution was removed, and cleaved-Caspase-3 primary antibody diluted at 1:1000 was applied directly onto the sections. The slides were placed in a humidified chamber and incubated overnight at 4°C (12–16 hours). The next day, after returning to room temperature, the slides were washed 3–5 times with 1× PBS on a shaker, each wash lasting 15 minutes. Subsequently, a fluorescently labeled rabbit anti-secondary antibody diluted at 1:5000 was applied and incubated for 1 hour at room temperature in the dark. The slides were then washed again under the same conditions with PBS in the dark, 3–5 times for 15 minutes each. Finally, nuclear counterstaining was performed using DAPI solution diluted at 1:2000, incubated for 1 hour at room temperature in the dark. Excess stain was removed by washing with PBS, and the sections were mounted with an anti-fade mounting medium. All representative images were acquired using a 20×objective lens on a Leica fluorescence microscope.

#### Paraffin sectioning and hematoxylin-eosin (H&E) staining

Zebrafish embryos from each group (20 embryos per group) were fixed overnight (12-16 hours) with 4% paraformaldehyde solution at 4°C. Following fixation, samples were dehydrated through a graded ethanol series: 70% ethanol (30 minutes), 80% ethanol (30 minutes), 95% ethanol (twice, 20 minutes each), and 100% ethanol (twice, 15 minutes each). The samples were then cleared in xylene (twice, 15 minutes each) and finally embedded by impregnation in molten paraffin at 60°C (three times, 1 hour each).

The embedded tissue blocks were continuously sectioned longitudinally at a thickness of 6 μm using a rotary microtome. The resulting sections were flattened in a 40°C warm water bath and mounted onto pre-treated glass slides. The sections were baked in a 60°C constant temperature oven for 2 hours to enhance tissue adhesion to the slides.

Prior to staining, the paraffin sections were sequentially deparaffinized in xylene (twice, 10 minutes each) and rehydrated through a descending ethanol gradient (100%, 95%, 80%, 70%; 2 minutes each), followed by a 5-minute rinse in PBS (pH 7.4). The staining procedure strictly adhered to the following protocol: hematoxylin staining for 2 minutes, rinsing under running tap water for 10 minutes for bluing, and counterstaining with 0.5% eosin alcohol solution for 30 seconds. After staining, the sections were rapidly dehydrated through an ascending ethanol series (70%, 80%, 95%, 100%; 30 seconds each), cleared in xylene (twice, 2 minutes each), and finally mounted with neutral resin.

All prepared sections were systematically examined under an optical microscope, with particular emphasis on evaluating cellular morphological changes and structural characteristics of specific organs such as the heart. Image acquisition was performed using a Leica microscope to ensure clear documentation of pathological alterations.

#### Determination of chloride ion (Cl^-^) concentration

For zebrafish embryos, 20 specimens each from the control and dLAN-treated groups were rinsed with pre-cooled PBS. Tissue homogenates were prepared using an electric homogenizer in an ice bath, with 10 μL of pre-cooled lysis buffer added per milligram of tissue. The resulting homogenates were centrifuged at 12,000 × g for 15 minutes at 4°C, and the supernatants were collected for analysis. A 50 μL aliquot of the tissue homogenate supernatant was thoroughly mixed with 250 μL of nitroprusside chromogenic agent, followed by a 10-minute incubation at 37°C under light-protected conditions. Absorbance was measured at 570 nm using a microplate reader. A standard curve was generated using NaCl standards (0–150 mmol/L), with three technical replicates per sample. The final chloride ion concentration was expressed as mmol per liter of tissue wet weight (mmol/L).

For mice, whole blood samples were collected via the tail vein. After standing for 30 minutes, the blood was centrifuged at 3,000 × g for 15 minutes at 4°C to separate the serum. A 50 μL aliquot of serum was mixed with 250 μL of nitroprusside chromogenic agent and processed following the same protocol for color development and measurement. Each assay batch included normal and high-value quality control samples, with the intra-assay coefficient of variation strictly controlled within 5%. Special attention was paid to avoiding hemolysis interference. The serum chloride ion concentration was ultimately expressed in mmol/L. To ensure methodological consistency, zebrafish and mouse samples were analyzed on the same microplate.

#### Mouse echocardiography

Following anesthesia induction, hair was thoroughly removed from the chest to the upper abdominal area using depilatory cream, and the skin was cleansed with a warm, moist gauze to prevent ultrasound signal interference. The mouse was then positioned in a supine posture on a pre-warmed imaging platform, with its limbs gently but securely immobilized using hypoallergenic medical tape to ensure full thoracic exposure while maintaining a natural anatomical position. To facilitate optimal acoustic transmission, a generous amount of sterile ultrasound coupling gel was applied to the electrode contact points on all four limbs and across the entire cardiac scanning area, with careful attention to eliminating minute air bubbles that could cause acoustic artifacts.

Image acquisition was performed using a linear array transducer with a central frequency of 30-40 MHz. For long-axis view imaging, the probe was positioned along the left sternal border with the orientation marker directed toward the mouse's head. The probe was then meticulously rotated approximately 45 degrees counterclockwise while fine-tuning its angulation and position to maintain complete skin contact until a clear two-dimensional long-axis image displaying the left ventricular outflow tract, mitral valve, and cardiac apex was visualized in B-mode. The system was subsequently switched to M-mode, and the sampling line was positioned perpendicularly at the mid-left ventricular cavity, just below the papillary muscle level, to acquire M-mode echocardiograms for quantitative analysis.

For short-axis view imaging, the probe was rotated approximately 90 degrees clockwise from the long-axis position while simultaneously adjusting the platform height and probe spatial orientation until a circular left ventricular cross-section with clearly visualized and symmetrically positioned papillary muscles was obtained in B-mode. Corresponding M-mode or two-dimensional measurements were then performed in this view.

Using images acquired through this standardized protocol, key cardiac functional parameters were precisely measured with integrated analysis software, including: left ventricular ejection fraction (EF%), left ventricular internal end-diastolic diameter (LVID/d), left ventricular internal end-systolic diameter (LVID/s), left ventricular posterior wall thickness at end-diastole (LVPW/d), and left ventricular posterior wall thickness at end-systolic (LVPW/s). This comprehensive approach enabled quantitative assessment of murine cardiac structure and systolic function. Measurements were taken over four consecutive cardiac cycles for each mouse.

### Quantification and statistical analysis

Statistical analyses were performed using GraphPad Prism 8.0 software. Normality of data distribution was assessed using the Shapiro–Wilk test. For comparisons between two groups, unpaired two-tailed Student's *t*-tests were applied when data passed normality; otherwise, the Mann–Whitney *U* test was used. For multiple-group comparisons, one-way ANOVA followed by Tukey's post hoc test was used. For datasets involving multiple related endpoints measured from the same animals—particularly the echocardiographic parameters in mice (EF, LVIDd, LVIDs, LVPWd, LVPWs; Figures 4H–4L) –Bonferroni correction was applied to control for Type I error across the two key comparisons (dLAN vs control and dLAN+bumetanide vs dLAN), resulting in a total of 10 comparisons and an adjusted significance threshold of *p< 0.005* (0.05/10). All data are presented as mean ± SEM. A *p<0.05* was considered statistically significant before correction, with adjusted thresholds clearly indicated for multiple comparisons. Exact sample sizes (*n*) and the specific statistical tests used for each figure panel are indicated in the corresponding figure legends.
