# Supplementary material for: Dim light at night induces cardiac injury in zebrafish embryos via disrupted chloride homeostasis
Source: iScience. 2026 Jun 3;29(6):115796. doi: 10.1016/j.isci.2026.115796 (PMC13255061; doi:10.1016/j.isci.2026.115796)
Supplement: Document S1. Figures S1–S7 and Table S1 [file mmc1.pdf]

iScience, Volume *29*

Supplemental information

Dim light at night induces cardiac injury in zebrafish embryos via disrupted chloride homeostasis

Risi Chen, Ying Zeng, Meixin Min, Ke Xu, Jia Wang, Tingting Liu, Yan Zhao, Weixin Zhang, Shuting Cheng, Yiyue Zhang, Chunru Wang, Dan Deng, and Xiaoping Xiao

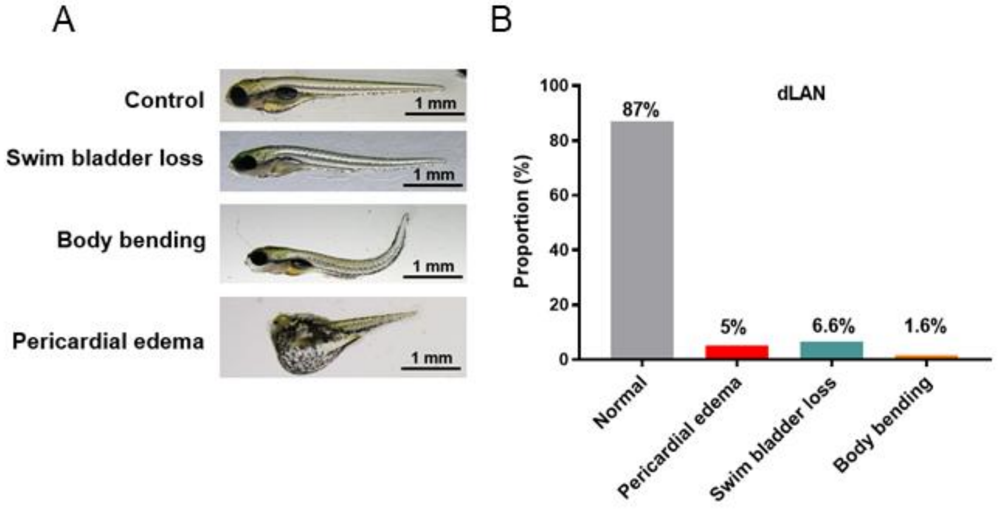

Figure S1. dLAN triggers systemic developmental abnormalities in zebrafish

(A) Representative images of 72 hpf zebrafish larvae exposed to dLAN (2.25 lux) versus control.

(B) Quantification of developmental defects.

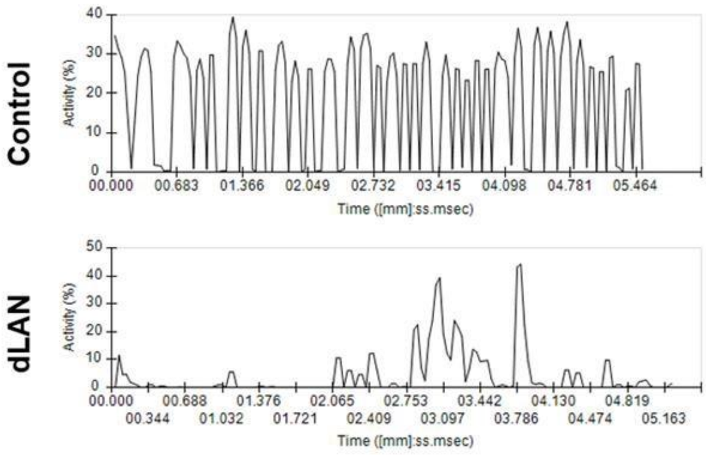

Figure S2. dLAN disrupts venous blood flow patterns

Representative blood flow velocity curves in the dorsal aorta analyzed by single-cell tracking DanioScope™.

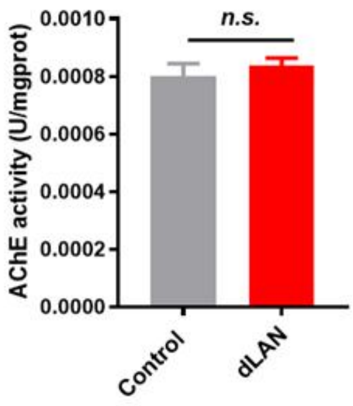

Figure S3. dLAN-induced locomotor deficit is independent of neuromuscular dysfunction

Acetylcholinesterase (AChE) activity assay in whole-larval homogenates (20 embryos/group). Absorbance measured at 412 nm (ΔOD/min/mg protein). No significant difference in AChE activity between dLAN and control groups, confirming cardiac-specific etiology of reduced mobility in Figure 1H. Data are represented as mean ± SEM. Statistical analyses using unpaired *t* test were performed on GraphPad Prism, *n.s.*= not significant.

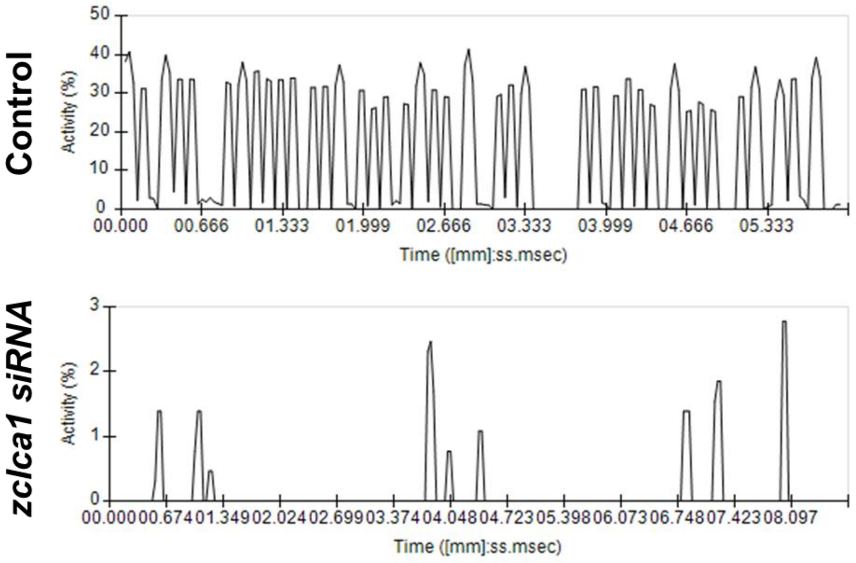

Figure S4. *zclca1* **knockdown recapitulates dLAN-induced hemodynamic impairment**

Representative blood flow velocity curves in *zclca1*-silenced zebrafish.

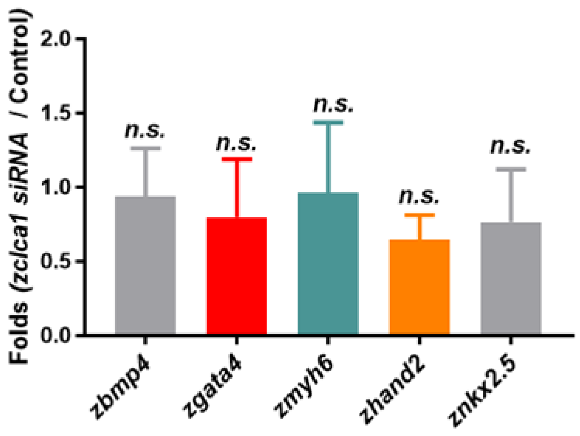

Figure S5. Cardiac developmental regulators are unaffected by dLAN or *zclca1* knockdown

qPCR analysis of cardiac transcription factors (*znkx2.5*, *zhand2*, *zgata4*, *zbmp4*, *zmyh6*) in *zclca1*-knockdown larvae. Data were normalized to *β-actin* and expressed as mean ± SEM. (n=3 independent experiments). Statistical analyses using unpaired *t* test were performed on GraphPad Prism, *n.s.*= not significant. Primer sequences can be found in the STAR Methods section.

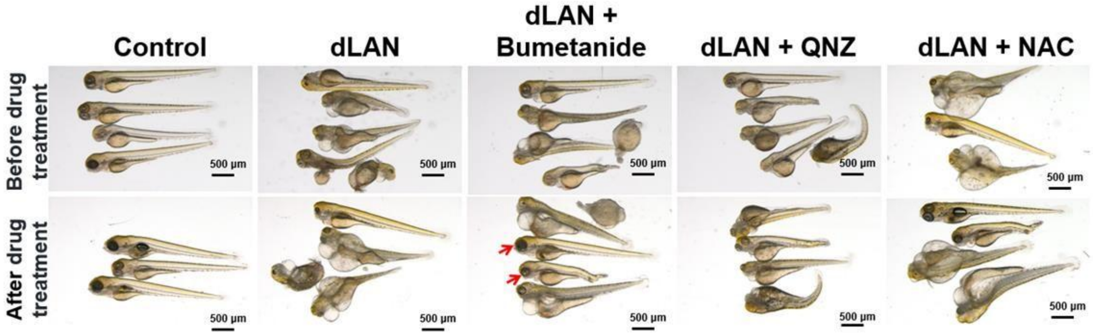

Figure S6. Therapeutic screening of antioxidants/anti-inflammatories for edema rescue

Representative images of zebrafish larvae with established dLAN-induced pericardial edema treated for 24 hr with bumetanide, QNZ (EVP4593), or NAC (N-acetylcysteine). Red arrows indicate responders showing reduced pericardial area.

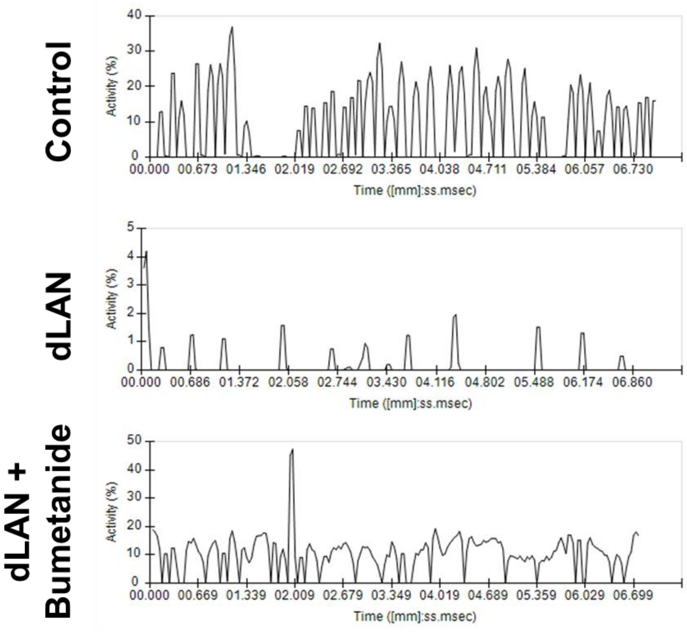

Figure S7. Bumetanide restores physiological blood flow dynamics

Blood flow velocity curves in dorsal aorta analyzed by DanioScope™.

Supplementary Table S1. Differential gene expression induced by dLAN

Function Group	Gene Name	Gene ID	Log_2 Ratio		
			dLAN 1 /Control 1	dLAN 2 /Control 2	dLAN 3 /Control 3
**ROS**	*zcyp2aa8*	ENSDARG00000104540	9.86556257004526	7.5482742663677	4.16058422572116
**ROS**	*zcyp2aa9*	ENSDARG00000098890	7.28096408444508	9.66237025360502	5.19658488537543
**ROS**	*zcyp2ad2*	ENSDARG00000021172	-3.35980730413246	-4.19288450413953	-3.5507269353906
**ROS**	*zcyp2x12*	ENSDARG00000092091	-6.46824968016676	-3.12247555952591	-3.1799428178608
**Ion channel**	*zclca1*	ENSDARG00000016290	-6.46824968016676	-6.24480518622216	-6.52116719927166
**Embryonic development**	*zhbbe3*	ENSDARG00000038147	6.74224750316492	3.06411513181559	6.05077523349676
**Embryonic development**	*zhoxb6b*	ENSDARG00000026513	6.7217821773919	6.86565822254679	6.43171744405937
**Cardiac failure**	*zcryaba*	ENSDARG00000042621	-4.08034016159005	-4.13997322986109	-5.08896514851505
**Cardiac failure**	*zela2*	ENSDARG00000056744	-3.05826812840894	-3.39634843251199	-5.63754719743444
**Cardiac failure**	*zela2l*	ENSDARG00000056765	-3.27587467781649	-3.61909811538061	-4.75351093543841
**Cardiac failure**	*zela3l*	ENSDARG00000007276	-3.02870317062075	-3.78953957329036	-5.16065193257273
**Cardiac failure**	*zgdf11*	ENSDARG00000044924	4.0946253027246	4.99239300791504	4.60113981579049
**Cardiac failure**	*zhp*	ENSDARG00000051890	3.03413058424524	4.03701823246884	5.97853934255846
**Cardiac failure**	*zhtra3a*	ENSDARG00000052895	6.85533501442637	3.91827658286124	5.39310114461371
**Cardiac failure**	*zmmp13a*	ENSDARG00000114451	9.28785987748915	4.72403889012328	7.37417638356142
**Cardiac failure**	*zmmp9*	ENSDARG00000042816	6.25906972899974	4.28137918363745	6.22217400558177
**Inflammatory**	*zlect2l*	ENSDARG00000033227	4.54389713099736	4.13770589293776	3.90206927028081
**Inflammatory**	*zlepb*	ENSDARG00000045548	4.85144740629908	6.01457535662501	8.65791726542557

**Inflammatory**	*zpla2g1b*	ENSDARG00000009153	-4.37267458403135	-3.18190971607962	-4.77279646155933
**Inflammatory**	*zsaa*	ENSDARG00000045999	9.16210374594012	5.21622659755908	6.99924436337744
**Inflammatory**	*zirg1l*	ENSDARG00000062788	5.60595166506522	4.93891928301598	7.33096637784221
**Inflammatory**	*zccr12b.1*	ENSDARG00000059410	3.76321050441194	4.03701823246884	4.07583981992623
**Inflammatory**	*zccr12b.2*	ENSDARG00000026417	4.31197266100508	4.4248946299427	5.03669815650425
**Inflammatory**	*zcmklrl2*	ENSDARG00000109654	5.62216389699087	5.78019202853706	4.04559631594103
**Inflammatory**	*zcsf3b*	ENSDARG00000098752	10.652361789244	6.28686800901833	10.8204601942348
**Inflammatory**	*zcxcl8a*	ENSDARG00000104795	7.21992055647789	3.98148486190154	6.35369157420235
**Inflammatory**	*zgpr84*	ENSDARG00000077308	8.41852793276462	4.8965827551904	6.55348599198874
**Inflammatory**	*zil11a*	ENSDARG00000037859	7.76055213111036	3.42406055670034	6.33354980762562
**Inflammatory**	*zil11b*	ENSDARG00000058557	6.95423377321269	7.12662931713177	8.24563934022073
**Inflammatory**	*zil1b*	ENSDARG00000098700	6.90247798695394	4.17124828435703	6.78923080155596
